# Supplementary material for: Granular Insights: Neutrophil Predominance and Elastase Release in Severe Asthma Exacerbations in a Pediatric Cohort
Source: Cells. 2024 Mar 18;13(6):533. doi: 10.3390/cells13060533 (PMC10969278; doi:10.3390/cells13060533)
Supplement: Supplementary file 1 [file cells-13-00533-s001.zip › cells-2886069-supplementary.pdf]

Supplementary Table S1. Patient Past History and Exposures

Variables	PICU (N)	ER (N)
Previous hospital admission to the floor (% 0)	25% (N = 16)	25% (N = 8)
Previous hospital admission to the ICU (% 0)	56% (N = 18)	75% (N = 8)
Systemic corticosteroid use (Median)	2x/yr (N = 17)	2.5x/yr (N = 7)
Smoke exposure (% Yes)	35% (N = 17)	28% (N = 7)
